# The Epidemiology of Human Immunodeficiency Virus (HIV) and Syphilis in Ghana: A Five-Year Single Urban Site Parallel Population-Based Analysis vis-à-vis the Sentinel Survey

**DOI:** 10.1155/2018/6574731

**Published:** 2018-05-30

**Authors:** James Osei-Yeboah, Sylvester Yao Lokpo, Francis Abeku Ussher, Verner Ndudiri Orish, Abdul-Wahab Mawuko Hamid, Mavis Puopelle Dakorah, Tibemponi Ntoni, Emmanuel Agbeko Nani, Felix Ayroe, Daniel Adigbli

**Affiliations:** ^1^Department of Medical Laboratory Sciences, School of Allied Health Sciences, University of Health and Allied Sciences, Ho, Ghana; ^2^Faculty of Health and Allied Sciences, Koforidua Technical University, Koforidua, Eastern Region, Ghana; ^3^Department of Microbiology and Immunology, School of Medicine, University of Health and Allied Sciences, Ho Volta Region, Ghana; ^4^Laboratory Department, St. Dominic Hospital, Akwatia, Eastern Region, Ghana; ^5^Laboratory Department, Ada-East District Hospital, Ghana Health Service, Ada, Greater Accra Region, Ghana; ^6^Laboratory Department, Ho Municipal Hospital, Ghana Health Service, Ho, Volta Region, Ghana; ^7^Laboratory Department, Krachi-West District Hospital, Ghana Health Service, Krachi, Volta Region, Ghana

## Abstract

The study was aimed at comparing the estimation of the burden and trends (2012–2016) of Human Immunodeficiency Virus (HIV) and Syphilis infections by the national Sentinel Survey vis-à-vis the use of population-based studies at a single urban site (Municipal Hospital) in Ho, the Volta Region of Ghana. Using blood donors as a proxy of the asymptomatic adult population, a retrospective analysis of secondary data on HIV and Syphilis testing was conducted using Ho Municipal Hospital's archives comprising 4,180 prospective blood donors. Published reports from the National Sentinel Survey for the Ho Sentinel Site comprising 2,452 pregnant women from 2012 to 2016 were used. The cumulative prevalence of HIV and Syphilis infections in the population-based survey was 4.78% and 2.58% while the epidemiology was estimated at 2.75% and 0.24% by the Sentinel Survey for the five-year under review. The new HIV and Syphilis infections were 3.78% and 2.46% in the population-based survey compared to 2.64% and 0.23% in the Sentinel Survey. Gender cumulative prevalence and the yearly trend was found to be higher in the general population compared to the pregnant women. The use of pregnant women to estimate the HIV and Syphilis epidemiology might not be representative of the general population.

## 1. Introduction

Human Immunodeficiency Virus (HIV) and Syphilis infections affect people disproportionately in many parts of the world [1, 2]. In the year 2016, an estimated 36.7 million people were living with HIV while the reported median prevalence of Syphilis was 1.11% worldwide [3, 4]. In Western and Central Africa, there were 6.1 million persons affected by HIV infection whereas an estimated prevalence of 3.04% Syphilis infection was reported in Africa, the highest by regional prevalence in 2016 [3, 4]. In Ghana, the national prevalence of HIV and Syphilis infections as determined by the 2016 HIV Sentinel Survey was estimated at 2.4% and 0.2%, respectively, with the Volta Region being one of the most HIV affected regions (2.7%) [5]. The clinical conditions associated with prolonged and untreated HIV and Syphilis infections may include weight loss, persistent generalized lymphadenopathy, chronic cough, diarrhea, recurrent fevers, tuberculosis, and oropharyngeal candidiasis, as well as infertility and adverse pregnancy outcomes, such as stillbirth [6]. There is lack of consensus in the scientific world regarding the choice of appropriate sample population to monitor the epidemiological patterns of HIV and Syphilis infections in the general population. While some have favoured the use of Sentinel Surveys in which pregnant women are recruited as proxy population because of easy accessibility and the relatively low cost of data collection, others are of the view that a population-based study in household surveys may be more representative of the general population largely due to the inclusion of men and people who fall outside the reproductive age brackets (15–49 years) [7–10]. In view of this, the UNAIDS/WHO Working Group on Global HIV/AIDS and STI Surveillance recommends that countries with generalized epidemic should conduct population-based studies periodically to validate the prevalence estimates of the annual HIV Sentinel Surveys [11]. Using blood donors as a proxy of the asymptomatic adult population, the current study aimed at comparing the estimation of the burden and trends (2012–2016) of HIV and Syphilis infections by the national Sentinel Survey vis-à-vis the use of population-based studies at a single urban site (Municipal Hospital) in Ho, the Volta Region.

## 2. Methods

### 2.1. Study Design and Study Site

We retrospectively analyzed secondary data of 4,180 prospective blood donors who visited the Ho Municipal Hospital from January 2012 to December 2016 and 2,452 pregnant women who were recruited into the annual HIV Sentinel Survey during the same period. The male and female donors included in this study aged between 18 and 58 years and their complete records were available for review. Variables including age, sex, and screening results of HIV and Syphilis assays were extracted from the archives at the blood bank of the Ho Municipal Hospital. The Ho Municipal Hospital serves as one of the four Sentinel Survey sites in the Volta Region. Published reports from the annual HIV national Sentinel Survey from 2012 to 2016 were retrieved where reports of HIV and Syphilis site-specific epidemiology (Ho Municipal Hospital HIV Sentinel Site) were extracted. The age of the pregnant women used in the Sentinel Survey ranged between 15 and 49 years.

### 2.2. Study Area

The Ho municipality is located in the Volta Region and serves as the capital of the Volta Region of Ghana. Ho is the capital of the municipality and the commercial hub of the entire Region. The total land size covers 2,361 square kilometers thus representing 11.5 percent of the region's total land area [12]. The total population is 192,871 with 94,951 males and 97,920 females and has a growth rate of 1.17% at the year 2010 [13].

### 2.3. Data Analysis

Data collected were entered into Microsoft Excel 2013 spreadsheet and validated for entry errors. Data were presented as frequencies and proportions. Differences between proportions and trends analysis were carried out using Fisher's exact test and chi-square test for trend where appropriate. A *p* value < 0.05 was considered as statistically significant. IBM Statistical Package for the Social Sciences (SPSS Inc. Chicago, USA; (http://www.spss.com/) version 22.00 was used for data analysis.

### 2.4. Ethical Consideration

Permission to carry out the study was obtained from the management of Ho Municipal Hospital. Ethical clearance for the study was granted by the Ethical Review and Scientific Committee of the School of Allied Health Sciences, University of Health and Allied Sciences, Ho (UHAS-SAHS-ERSC:029A/2017). Analysis of the data was anonymous and nonlinked, and no donor names were retrieved from the archives.

## 3. Results

During the five years under review, the cumulative prevalence of HIV among the 4,181 potential blood donors at the Ho Sentinel site was estimated at 4.78%. Within the same period, the cumulative prevalence of HIV by the Sentinel Survey was estimated at 2.45% (*p* < 0.0001). The estimated prevalence of HIV at each year of the review was found to be higher among the blood donor population than the estimated prevalence by the Sentinel Survey, though significant statistical difference was observed for only 2012 and 2013. While the infection rate stayed fairly stable, above 4.00% among the blood donor population (*p* for trends = 0.0848) with the exception of 2015, an undulating trend in prevalence was observed among the Sentinel population, peaking in 2014 (4.37%) and troughing in the previous year (0.60%) (See Table 1).

The cumulative burden of Syphilis among the 4,181 potential blood donors at the Ho Sentinel site was estimated at 2.58%. The cumulative burden of Syphilis among pregnant women by the Sentinel Survey within the same period was found to be significantly lower (0.24%, *p* < 0.0001). Except in 2014, the year-on-year infection rate was observed to be significantly higher among the blood donor population above 2% compared to the rate estimated among the Sentinel population (See Table 2).

Using the rate of HIV infection among the age group of 15–24 years as a proxy of new infections, a total of 46 new infections representing 3.78% of the 1,218 blood donors within that age group were recorded within the five years under review. New infections in the Sentinel Survey for the five-year period stood at 23 out of 870 participants representing 2.64% new HIV infection rate. The last three years of the Sentinel Survey recorded a rise in new HIV infections, peaking in 2014 (5.45%), while the last two years of the donor screening review saw a fall in the rate of new HIV infections (See Table 3).

Using the donor screening records and the age group of 15–24 year as a proxy of new Syphilis infections, a total of 30 new infections, representing 2.46% of the 1,218 blood donors within that age group, were recorded within the five years under review. New infections in the Sentinel Survey for the five years period stood at 2 out of the 870 participants, representing 0.23% new Syphilis infection rate. A steady trend of new Syphilis infection over the period under review was observed among donors, at above 2% except in 2014 (See Table 4).

**Figure 1:**
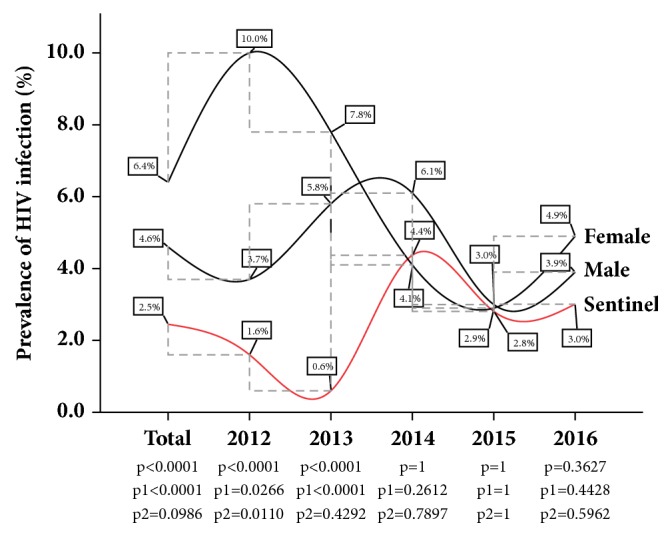
Year-on-year gender-specific HIV prevalence parallel analysis with the Sentinel Survey at the Ho Sentinel site: *p* compares female donor and Sentinel, *p*1 compares male donor and Sentinel, and *p*2 compare female donor and male donor. *p* is significant at 0.05.

The cumulative five-year burden of HIV infection among the female donor population was 6.40% and 4.60% among the male donor population. In comparison with the cumulative rate of HIV infection among the Sentinel population, the cumulative rate of infection among both female and male was found to be significantly higher (*p* < 0.0001). Though not statistically significant for the last three years of the review period, both the female and the male donor population presented a higher HIV infection rate compared to the estimates from the Sentinel Survey. However, 2014 backed this trend, where the Sentinel estimate (4.40%) was slightly above the female donor screening estimate (4.10%). The year 2015, marking the trough of the year-on-year HIV infection for both the female (2.90%) and the male (3.00%) donor populations, was also observed as the year with the closest rate of HIV infection to the Sentinel Survey estimate (2.80%) (See Figure 1).

**Figure 2:**
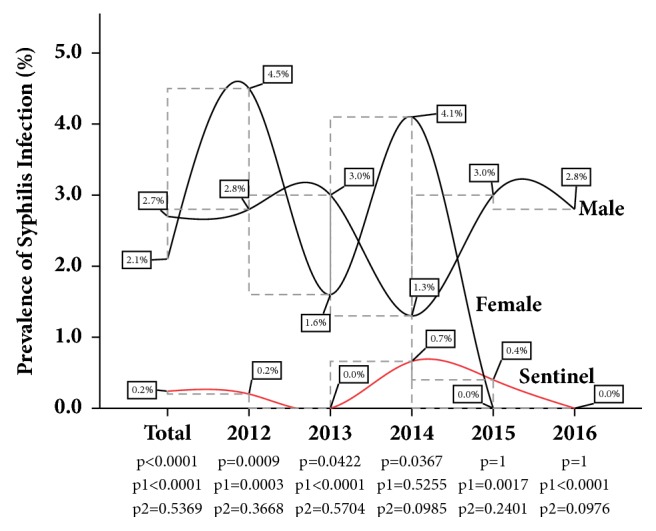
Year-on-year gender-specific Syphilis parallel analysis with the Sentinel Survey at the Ho Sentinel site: *p* compares female donor and Sentinel, *p*1 compares male donor and Sentinel, and *p*2 compare female donor and male donor. *p* is significant at 0.05.

The cumulative five-year burden of Syphilis infection among the female donor population was 2.10% and 2.70% among the male donor population. In comparison with the cumulative rate of Syphilis among the Sentinel population (0.20%), the cumulative rate of infection among both female and male was found to be significantly higher (*p* < 0.0001). In general, the male donor population significantly presented with a higher year-on-year Syphilis infection rate compared to the Sentinel population, for the period under review. The female donor population recorded significantly higher Syphilis infection rate compared to Sentinel estimates in the first three years of the review period. However, no Syphilis infection was recorded among the female donor in the last two years of the review (See Figure 2).

Among the Sentinel population, the five-year burden of HIV infection crosses the 3% rate only among the 20–24 year (3.25%) and the 40–44 year (3.90%) groups. Except in the lower age category (15–19 year), the epidemiology density of HIV was found to be over and above 3% rate irrespective of age and gender in the blood donor population. The five-year burden of HIV infection peaked among the 35–39 year group for both the total donor population and the male donor subgroup and peaked among the 40–44 year group for the female donor subpopulation (See Table 5).

Among the Sentinel population, the five-year burden of Syphilis infection was highest among the 35–39 year group. The epidemiology density for Syphilis infection in the five years under review was found to cluster at the higher (45–49 years) and lower (15–19 years) extreme of the age categorization both the total donor population and the male donor subgroup and only in the higher extreme age group (45–49 year) among the female donor subpopulation (See Table 6).

## 4. Discussion

In the current study, we sought to compare the burden and five-year (2012–2016) trend of HIV and Syphilis infections using blood donors as a proxy for an adult asymptomatic population-based surveillance and pregnant women in the Sentinel Survey among Ghanaians at a single urban site (Municipal Hospital) in the Volta Region of Ghana. The cumulative prevalence of HIV and Syphilis infections among the 4,181 blood donors during the five-year period was estimated at 4.78% and 2.58%, respectively, while in the Sentinel Survey, the prevalence was 2.75% and 0.24%, respectively, for HIV and Syphilis infections among the 2,452 recruited pregnant women who formed part of the national survey for this Sentinel site. In general, the estimated yearly prevalence of HIV and Syphilis infections was found to be significantly higher in the population-based study compared to that recorded in the Sentinel Survey, except in the years 2014, 2015, and 2016 where the HIV burden between the two surveys was statistically comparable (Tables 1 and 2). Our results suggest that asymptomatic adult participants in the general population are more likely to be infected with blood-borne pathogens in comparison with women in the Sentinel Survey in the study catchment area. The relatively low rate of infectious burden observed in the Antenatal Clinic (ANC) attendees in the Sentinel Survey could indicate that the use of this targeted population (pregnant women) as a proxy for the general population in the study area (Ho municipality) could underestimate the infectious burden. Earlier studies conducted in other African jurisdictions have reported findings consistent with the current results. Fylkesnes et al. [14] in Zambia, Glynn et al. [15] in Cameroon, Changalucha et al. [16] in Tanzania, and Gonese et al. [7] in Zimbabwe have all reported a lower blood-borne infection rate among ANC attendees compared to individuals sampled from the general population. Although the information in the current work is limited in precisely explaining the discordance in the prevalence rates between the two populations, various postulations have been suggested by some authors to account for this phenomenon. According to Gonese et al. [7], ANC surveillance data exclude men and nonpregnant women in the general population who may have the infections thus rendering ANC data imprecise representatives of the general population. In their work in Tanzania, Changalucha et al. [16] posited that pregnant women attending ANC services would have a different socioeconomic background and they may be more cautious with their health issues compared to their peers in the general population. In the opinion of Fylkesnes et al. [14], pregnant women who seek ANC services elsewhere, for example, traditional healers or private clinics rather than ANC Sentinel sites, would not be factored into computing for the overall infectious burden in the entire population.

In contrast, some studies have reported a close agreement in the estimated prevalence of blood-borne infections between population-based studies and ANC surveillance systems while others observed a lower prevalence of the infections within the general population compared to pregnant women in the Sentinel Survey [7–9, 17]. The reasons adduced to explain the comparable results of prevalence estimates between population-based surveys and Sentinel Surveys include the restriction of comparison to individuals living within 15 km radius of ANC surveillance sites, simultaneous standardization of sociodemographic distribution between the two surveys, and adjusting for parity using correction factors [7, 8].

The estimation of HIV and Syphilis new infections in a defined population is an essential component of population-based studies and ANC surveillance systems. Individuals in the age group 15–24 years are considered a proxy measure for new infections (incidence), owing to their early sexual debut and to the fact that infections in this age category are assumed to be recently acquired [18]. In the present study, the incidence of HIV and Syphilis was estimated at 3.78% and 2.46%, respectively, in the population-based study whereas in the Sentinel Survey, incidence of 2.64% and 0.23%, respectively, for HIV and Syphilis was reported within the five-year review period. Also, the estimated yearly burden of HIV and Syphilis new infections was in general found to be significantly higher in the population-based study when compared to the Sentinel Survey during the period under review (Tables 3 and 4). The current result highlights the high rate of sexually transmitted pathogens among the relatively young Ghanaian population in the study area. Factors proposed for young adults vulnerability to acquiring sexually transmitted infections include early sexual debut, education level attainment, multiple sexual partners, poor relationship with parent, having unprotected sex, and low economic status [19, 20].

In the present study, when participants in the population-based study were stratified according to gender, the cumulative HIV and Syphilis infection rates, as well as the year-on-year trend analysis among both female and male subpopulations, were generally found to be significantly higher than those observed in the Sentinel Survey (Figures 1 and 2). The results compare favourably with those reported previously, where surveillance data of ANC tended to underestimate the epidemiology in women and the overall infectious burden (men and women) in the general population [7, 14–16]. According to Montana et al. [8], women with HIV infection may be physiologically less likely to become pregnant and therefore access ANC services; hence the use of ANC data could lead to underrepresentation of nonpregnant women of the same age in the general population.

One of the significant concerns about the representativeness of ANC surveillance data in prevalence estimates is that ANC data tends to overestimate the infectious burden among the young people and underestimate among the older generation compared to women of the same age in the general population, a phenomenon which is often attributed to selection bias [7, 8]. Contrary to this view, our study showed that there was a general underestimation of HIV and Syphilis infection rate among the various age groups in the Sentinel Survey in comparison to participants in the population-based study (Tables 5 and 6). The difference in our results from those reported previously by Montana, et al. [8] and Changalucha et al. [16] where the prevalence estimates of HIV were higher in the youngest age group (15–19 years) among ANC attendees than in women from the general population could be due to the difference in sample population. The two previous studies recruited community members and participants in a Demographic and Health Survey respectively compared to blood donors in the current study. However, the drawback of using household surveys to estimate the infection rates which includes bias due to nonresponse and exclusion of nonhousehold-based populations, as well as the high cost in conducting such surveys annually, have been well documented [7]. Blood donors, on the other hand, could be more representative (includes men and women) than using only pregnant women and cheaper to recruit (compared to household surveys) and the issue of nonresponse could be nonexistent since blood donors are already volunteers to testing. The appropriateness of the use of blood donors as representative Sentinel group has also been corroborated by Borgdorff et al. [21], but they suggested that, in such surveys, data such as age, sex, and location should be standardized. However, the limitation in the use of blood donors as a proxy of the population is the suggestion that blood donors may exhibit a healthier lifestyle compared to the general population and this may lead to a lower rate of infections in such groups than would be in the general population [22].

## 5. Conclusion

Our study has demonstrated that the Sentinel Survey underestimated HIV and Syphilis prevalence rates compared to a population-based study population. This suggests that using pregnant women to estimate the HIV and Syphilis epidemiology might not be representative of the general population.

## Figures and Tables

**Table 1:** Year-on-year prevalence of HIV infection among blood donors and pregnant women (Sentinel survey) in the Ho municipality.

Parameter	Total	2012	2013	2014	2015	2016	*p* for trends
*Donors*	4,181	886	1,127	630	646	892	
Positive	200 (4.78)	40 (4.51)	68 (6.03)	37 (5.87)	19 (2.94)	36 (4.04)	0.0848
*Sentinel*	2,452	500	497	458	499	498	
Positive	60 (2.45)	8 (1.60)	3 (0.60)	20 (4.37)	14 (2.81)	15 (3.01)	0.0217
*p*-value	<0.0001	0.0035	<0.0001	0.3348	1	0.3742	

Data is presented as frequency with the corresponding percentage in parenthesis. *p* is significant at 0.05.

**Table 2:** Year-on-year prevalence of Syphilis infection among blood donors and pregnant women in the Ho municipality.

Parameter	Total	2012	2013	2014	2015	2016	*p* for trends
*Donors*	4,181	886	1,127	630	646	892	
Positive	108 (2.58)	27 (3.05)	32 (2.84)	10 (1.59)	17 (2.63)	22 (2.47)	0.3874
*Sentinel*	2,452	500	497	458	499	498	
Positive	6 (0.24)	1 (0.20)	0 (0.00)	3 (0.66)	2 (0.40)	0 (0.00)	0.9989
*p*-value	<0.0001	<0.0001	<0.0001	0.2579	0.0038	<0.0001	

Data is presented as frequency with the corresponding percentage in parenthesis. *p* is significant at 0.05.

**Table 3:** Year-on-year prevalence of new HIV infection (15–24 years) among blood donors and pregnant women in the Ho municipality.

Parameter	Total	2012	2013	2014	2015	2016	*p* for trends
*Donors*	1,218	243	337	194	185	259	
Positive	46 (3.78)	10 (4.12)	17 (5.04)	10 (5.15)	3 (1.62)	6 (2.32)	0.0678
*Sentinel*	870	178	207	165	160	160	
Positive	23 (2.64)	1 (0.56)	1 (0.48)	9 (5.45)	7 (4.38)	5 (3.13)	0.0147
*p*-value	0.1923	0.0286	0.0025	1	0.1974	0.7549	

Data is presented as frequency with the corresponding percentage in parenthesis. *p* is significant at 0.05.

**Table 4:** Year-on-year prevalence of new Syphilis (15–24 years) infection among blood donors and pregnant women in the Ho municipality.

Parameter	Total	2012	2013	2014	2015	2016	*p* for trends
*Donors*	1,218	243	337	194	185	259	
Positive	30 (2.46)	5 (2.06)	12 (3.56)	2 (1.03)	4 (2.16)	7 (2.70)	0.8932
*Sentinel*	870	178	207	165	160	160	
Positive	2 (0.23)	0 (0.00)	0(0.00)	1 (0.61)	1 (0.63)	0 (0.00)	0.5478
*p*-value	<0.0001	0.0760	0.0046	1	0.3780	0.0473	

Data is presented as frequency with the corresponding percentage in parenthesis. *p* is significant at 0.05.

**Table 5:** Distribution of five-year burden of HIV infection by age categories.

Parameter	15–19	20–24	25–29	30–34	35–39	40–44	45–49
Sentinel	4 (1.40)	19 (3.25)	12 (1.76)	14 (2.82)	8 (2.56)	3 (3.90)	0 (0.00)
Total	5 (2.84)	41 (3.93)	62 (4.85)	27 (4.22)	36 (7.68)	15 (5.38)	14 (4.71)
Female	1 (3.03)	8 (6.61)	8 (5.88)	7 (6.48)	3 (7.14)	3 (11.11)	1 (5.56)
Male	4 (2.80)	33 (3.58)	54 (4.73)	20 (3.76)	33 (7.73)	12 (4.76)	13 (4.66)

*p*-value	0.3120	0.5837	0.0004	0.2617	0.0023	0.7733	1
*p*1-value	0.4239	0.1120	0.0101	0.0774	0.1294	0.1791	1
*p*2-value	0.4499	0.7739	0.0007	0.4859	0.0030	1	1
*p*3-value	1	0.1300	0.5266	0.1942	1	0.1672	0.5917

Data is presented as frequency with the corresponding percentage in parenthesis. *p* is significant at 0.05. *p* compares total and sentinel, *p*1 compares Female and Sentinel, *p*2 compares male and sentinel, and *p*3 compares female and male.

**Table 6:** Distribution of five-year burden of Syphilis infection by age categories.

Parameter	15–19	20–24	25–29	30–34	35–39	40–44	45–49
Sentinel	0 (0.00)	2 (0.34)	1 (0.15)	1 (0.20)	2 (0.64)	0 (0.00)	0 (0.00)
Total	7 (3.98)	23 (2.21)	32 (2.50)	12 (1.88)	15 (3.20)	2 (0.72)	17 (5.72)
Female	0 (0.00)	4 (3.31)	2 (1.47)	1 (0.93)	2 (4.76)	0 (0.00)	1 (5.56)
Male	7 (4.90)	19 (2.06)	30 (2.63)	11 (2.07)	13 (3.04)	2 (0.79)	16 (5.73)

*p*-value	0.0011	0.0025	<0.0001	0.0091	0.0215	1	1
*p*1-value	1	0.0093	0.0732	0.3254	0.0703	1	1
*p*2-value	0.0004	0.0055	<0.0001	0.0064	0.0313	1	1
*p*3-value	0.3503	0.33	0.5686	0.7012	0.6349	1	1

Data is presented as frequency with the corresponding percentage in parenthesis. *p* is significant at 0.05. *p* compares total and Sentinel, *p*1 compares females and Sentinel, *p*2 compares male and Sentinel, and *p*3 compares female and male.

## Data Availability

The data used for the current study could be acquired through an e-mail request to the corresponding author without any restrictions.
